# Prediction of treatment efficacy for prostate cancer using a mathematical model

**DOI:** 10.1038/srep21599

**Published:** 2016-02-12

**Authors:** Huiming Peng, Weiling Zhao, Hua Tan, Zhiwei Ji, Jingsong Li, King Li, Xiaobo Zhou

**Affiliations:** 1 Division of Radiologic Sciences – Center for Bioinformatics and Systems Biology, Wake Forest School of Medicine, Medical Center Boulevard, Winston-Salem, NC 27157, USA; 2 College of Biomedical Engineering and Instrument Science, Zhejiang University, Hangzhou, China

## Abstract

Prostate immune system plays a critical role in the regulation of prostate cancer development regarding androgen-deprivation therapy (ADT) and/or immunotherapy (vaccination). In this study, we developed a mathematical model to explore the interactions between prostate tumor and immune microenvironment. This model was used to predict treatment outcomes for prostate cancer with ADT, vaccination, Treg depletion and/or IL-2 neutralization. Animal data were used to guide construction, parameter selection, and validation of our model. Our analysis shows that Treg depletion and/or IL-2 neutralization can effectively improve the treatment efficacy of combined therapy with ADT and vaccination. Treg depletion has a higher synergetic effect than that from IL-2 neutralization. This study highlights a potential therapeutic strategy in effectively managing prostate tumor growth and provides a framework of systems biology approach in studying tumor-related immune mechanism and consequent selection of therapeutic regimens.

Prostate cancer (PCa) is the most commonly diagnosed non-skin malignancy and the second leading cause of cancer-related deaths in American men^1^. The main treatment modalities for prostate cancer include surgery, radiotherapy and hormone therapy. Androgen deprivation therapy (ADT) is an anti-hormone therapy and used to control prostate cancer cell growth by suppressing or blocking production/action of androgens in men. Unfortunately, a significant number of primary PCa patients treated with ADT eventually develop incurable castration-resistant disease^2^. The possible mechanism of resistance is due to a switch of tumor cells from androgen-dependent or castration-sensitive prostate cancer (CSPC) to androgen-independent or castration-resistant prostate cancer (CRPC), and the CRPC cells do not require normal levels of androgen for supporting tumor cell growth^3^,^4^,^5^.

The tumor microenvironment contributes to tumor initiation and progression^6^. During tumor development, tumor and its microenvironment modulate immune cells towards a pro-tumorigenic phenotype and establish an immune suppressive niche, therefore facilitate tumor growth and metastasis. The molecular and cellular nature of the tumor immune microenvironment has impact in tumor development by altering the balance of suppressive versus cytotoxic responses in the vicinity of tumors. Thus, immunotherapy has a daunting task in a host with an established cancer^7^,^8^. Recent studies have suggested a potential synergy between immunotherapy (vaccination) and androgen ablation^9^. Androgen deprivation showed that removal of androgen in male mice increased lymphopoiesis, renewed thymopoiesis, and enhanced immune responses^10^,^11^. These data suggest that it would be beneficial to prostate cancer patients using combined therapeutic immunotherapy with androgen deprivation. The presupposition for gaining maximum benefit using combined therapies is a profound understanding of the impact of androgen ablation on the tumor-associated immune system, which will contribute to designs of more effective therapeutic regimens for patients with advanced PCa.

To study the systematic effect of ADT to tumor-associated immune responses, *in vivo* animal models that recapitulate the nature of human PCa are needed. Therefore, we have developed a prostate-specific Pten^−/−^ mouse model and data analysis showed that this mouse model mimicked the scenario of progressive PCa well^12^. Both effector (CTLs: cytotoxic T lymphocytes) and inhibitory (Tregs: regulatory T cells) immune mechanisms were amplified by surgical castration (ADT)^13^,^14^,^15^. In the prostate-specific Pten^−/−^ mouse model, our colleagues found that ADT resulted in apoptotic death of cancerous prostate epithelium and the antigens shed by the dying prostate tumors increased the function of CTLs^15^. The activation of CTLs was following by an induction of IL-2 and expansion of Treg, which led to the inhibition of CTLs in the prostate draining lymph nodes^15^. These data indicated that although the immune response from the effector cells were augmented by castration in the Pten^−/−^ mice, the concomitant secretion of IL-2 and expansion of Tregs as two major types of immune inhibitory brakes were responsible for a short-term, but not persistent increase of immune response following ADT. Therefore, depletion of Treg or neutralization of IL-2 has potentials in enhancing therapeutic efficacy of combined therapy with ADT and immunotherapy for PCa.

Mathematical modeling is a description of a system using mathematical concepts and can provide a powerful approach for simulating a complicated system, such as interaction of tumor cells with their environment. Such comprehensive study will enhance our understanding of tumor dynamics and develop new approaches/strategy for optimization of existing therapies. The systems modeling approaches have been widely used for quantitatively understanding complex biological systems (see^16^,^17^,^18^,^19^ and reference therein). Several mathematical models have been used to quantify ADT^5^,^20^,^21^,^22^,^23^ or immunotherapy^24^,^25^,^26^ in prostate cancer. Although systems modeling approaches have not been applied for assessing efficacies of combined therapies with ADT and vaccines for prostate cancer, some approaches have been developed previously for the combined therapies in prostate cancer as well as in bone metastatic prostate cancer such as the combined ADT and radiation therapy or chemotherapy^27^,^28^. Moreover, the combined therapy of ADT and immunotherapy has recently also been implicated as a promising therapy for prostate cancer from a biological point of view^9^,^29^. Therefore, it is significant to apply systems modeling approaches to explore the combined therapies of ADT and vaccines.

In this study, we developed a novel mathematical model to characterize the effect of ADT on immune system and the efficacy of combined therapy with ADT and vaccines. We used a system of ordinary differential equations (ODEs) to describe dynamics of individual components as well as interactions between components in the model system. Our model system consisted of multiple components, such as androgen, CTLs, Tregs, IL-2, CSPCs and CRPCs. Androgen and CSPCs were used to model ADT effect and Treg and IL-2 for the inhibitory effect of immune system. T cells are the key cells of immune system. CTL can recognize an antigen on tumor cells via T cell receptor (TCR) and kill the tumor cells. Tregs are key mediators of tumor immune suppression and elevated numbers of Tregs have been associated with all of major cancers. Naïve T cells must interact with dendritic cells (DCs), the professional antigen-presenting cells of the immune system, to become activated. There are two types of DCs in lymphoid and they are distinguished based on their functions^26^,^30^. IL-2 secreted by CTL leads to expansion of Tregs and subsequent inhibits CTL function^13^,^14^,^15^. Thus, a novel multi-scale and multi-compartment model was developed for addressing the interaction of ADT and immune system based on the published ADT or immunotherapy modeling approaches^5^,^20^,^21^,^22^,^24^,^25^,^26^. Two compartments used in this model were prostate and lymphoid^31^,^32^. The model inputs were defined by therapeutic strategies, individuals or combinations. The model outputs were defined by tumor burdens, both instantaneous and average over a period of time after treatment. The outcome predictions for multiple treatments were achieved by training the parameterized model with *in vivo* experimental data under several treatment conditions in our mouse model. It is worth noting that the basic idea of the whole mathematical strategy applied in this study is simple. We used a few known input-output data from animal studies to infer a quantitative system and then changed input to predict unknown output from this system. Finally, the model-based predictions were used to verify the proposed hypothesis that Treg depletion and/or IL-2 neutralization are capable to improve the treatment efficacy of combined therapy with ADT and immunotherapy. Most importantly, our predicted data using the modeling system indicated that Treg depletion had a better effect than that by IL-2 neutralization. Overall, this study highlights treatment approaches for effectively reducing castration-resistant tumor burden of prostate cancer and provides a framework of systems modeling approach for studying tumor-related immune responses.

## Results

### General mathematical model for predicting therapeutic outcomes in treatment of prostate cancer

**Figure 1:**
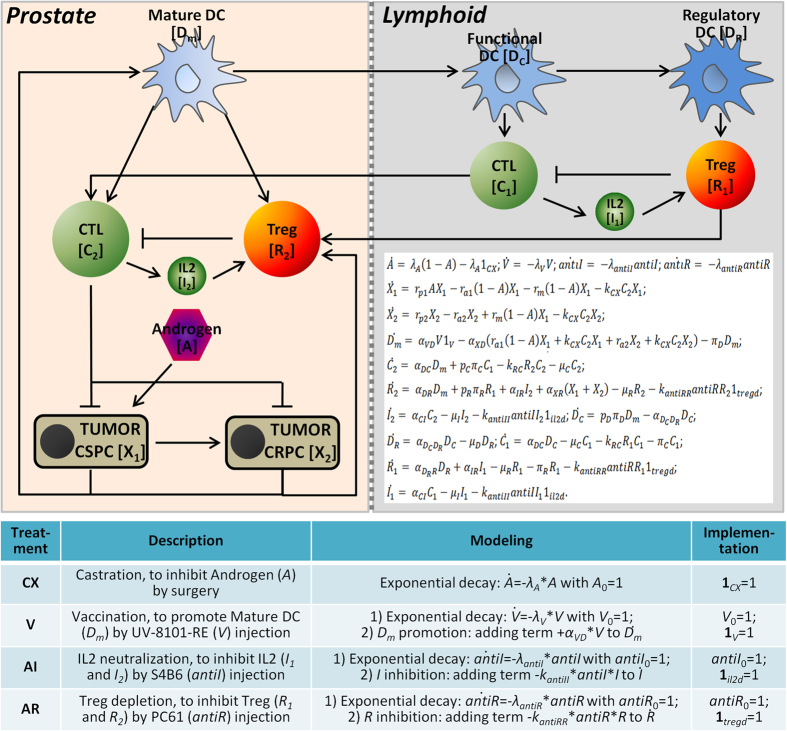
Model construction for predicting treatment outcomes of prostate cancer. The upper panel shows the interaction diagrams between tumor cells and the immune microenvironment. The prostate tumor includes castration-sensitive prostate cancer (CSPC) and castration-resistant prostate cancer (CRPC) in prostate compartment and the tumor-related immune system consists of dendritic cells (DCs), cytotoxic T cells (CTLs) and T regulatory cells (Tregs) in both prostate compartment and lymphoid compartment. The arrow and hammer head represent promotion and inhibition in the interaction, respectively. Mathematical formulas (15 ordinary differential equations) in the right corner of upper panel are used to describe the dynamics of the system. The first four formulas are used for modeling four types of treatments included in this study, i.e., castration (CX), vaccination (V), IL-2 neutralization (AI) and Treg depletion (AR). The definition and modeling procedure of four treatments are shown in the table of lower panel. In the column of implementation, both of the initial value and indicator function are given with value 1 when the corresponding treatment is implemented, otherwise both of the values are 0, except for the initial value of Androgen which is given a value 1 (*A*_0 _= 1) for all cases in the model system.

We developed an ODE-based mathematical model to predict treatment outcomes for prostate cancer as shown in Fig. 1 (also see the Methods). The flowchart displayed in Fig. 1 shows modeling components, including dynamics of the disease progression following androgen stimulation and actions of various immune cells. Time-associated interplays between tumor and immune cells and the effects of different treatment strategies were also taken into consideration. Two types of cancer cells, androgen-dependent/castration-sensitive prostate cancer cell (CSPC) and androgen-independent/castration-resistant prostate cancer cell (CRPC), were included in this model^3^,^4^,^5^. Androgen functions as a stimulus to the growth of CSPC, however deprivation of androgen can induce CSPC into an androgen-independent phenotype. Immune system is an important component in the tumor microenvironment. Interplay between tumor cells and host immune cells is very complicated. In our model, we simplify the immune response by focusing on several key components of immune system. Lymphoid compartment is used as a representative of the prostate-draining lymph nodes and spleen, and blood vessel compartment for tumor-immune communication between prostate compartment and lymphoid compartment was not modeled for simplicity. In the modeling system, CTLs represent effector immune function and both Tregs and IL-2 represent inhibitory effect of immune system. The ODEs employed in the modeling system are shown in Fig. 1 and detailed descriptions of these equations displayed in the section of Methods and Supplementary data. This model included four types of basic treatments, including androgen deprivation therapy or castration (CX), immunotherapy/vaccination with UV-8101-RE to induce CD8^+^ T-cell response (V), IL-2 neutralization (AI) and Treg cell depletion (AR). We used an exponential decay formula to model CX. One additional variable was introduced for modeling the outcomes from other three treatments, in which the drug effects were accompanied by an exponential decay. We also use indicator functions to simulate tumor responses to the treatments. Details are shown in the lower panel of Fig. 1 (also see the section of Methods as well as Supplementary Text S1).

**Figure 2:**
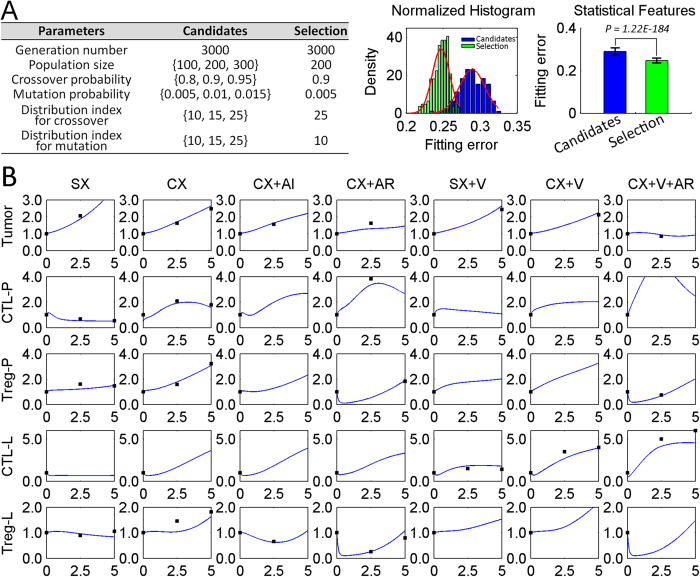
GA parameter selection and data fitting. (**A**) GA parameter selection in MGA. The left table shows the candidates and selection for GA parameter settings and the two subfigures on the right display the comparisons of fitting errors before and after parameter selection. The normalized histograms of fitting errors are based on 243 data points (the number of candidates of GA parameter sets) for candidate case and 500 data points (the number of replicates of parameter estimation based on selected GA parameters) for selection case. The p-value was calculated by unpaired and two-tailed student’s t-test. (**B**) Data fitting. The blue lines show the predicted results and black dots are data points collected from experimental data. Horizontal rows include five types of variables (“-P” represent in prostate and “-L” in lymphoid) and vertical columns embrace seven types of experimental conditions. In each subplot, the y-axis represents relative number of cell population and x-axis represents the time in weeks post-treatment. Note that the plot presents all the variables including those whose time series data were even not collected or incomplete for simplicity purpose of presentation, and the initial conditions, which have all been set to 1, were not included in the calculation of error during parameter fitting.

To parameterize the model, dynamical cell population data obtained from seven experimental conditions were used (see Methods for details about the data). In general, tumor weight and populations of CTLs and Tregs in prostate tissues and prostate-draining lymph nodes of prostate-specific Pten^−/−^ mice were measured at different time points under different experimental conditions. A modified Genetic Algorithm (MGA) was employed to estimate unknown parameters in our ODEs system by taking advantage of high performance computing capacity in Texas Advanced Computing Center (TACC). The objective function minimized by MGA was defined as the sum of squared errors between simulation data and experimental data in all of seven conditions. MGA is an improved implementation of GA with an additional procedure of GA parameter selection (see Methods for details). The goodness of GA parameter selection is shown in Fig. 2A. The left table shows the candidates of GA parameter settings, including total 243 possible parameter sets, as well as the selected settings with the best estimation performance. We compared the fitting errors between candidate group and selected GA parameter settings. The candidate group and selected group had 243 and 500 errors, respectively. The selected group was obtained through 500 times repeated estimation with random initial seeds in GA while other GA parameters were fixed with selected GA parameter settings. Our analysis showed that the performance of parameter estimation had a statistically significant improvement with GA parameter selection. With the selected GA parameter settings, the parameter estimation was re-evaluated 500 times and then an optimized parameter set was achieved (see Supplementary Table S2 for details). Fig. 2B shows the fitting results between experimental and simulation data using our quantitative model with optimized parameters. The dynamics of other variables in the modeling system are presented in Fig. S2. In general, a good fitting performance between experimental and simulation data suggests that the proposed algorithm for parameter estimation is effective in training big ODEs systems. The results in Fig. 2B show that our established model is capable to reproduce cellular dynamics for all of seven treatment conditions simultaneously, suggesting our established model is powerful in predicting the outcomes from combined therapies. Parameter identifiability analysis was conducted by calculating coefficients of variation (CVs) of model parameters (see Methods for details) and only one out of 25 estimated model parameters was non-identifiable (Fig. S3 and Table S2).

### Evaluation of model performance using cross-validation

**Figure 3:**
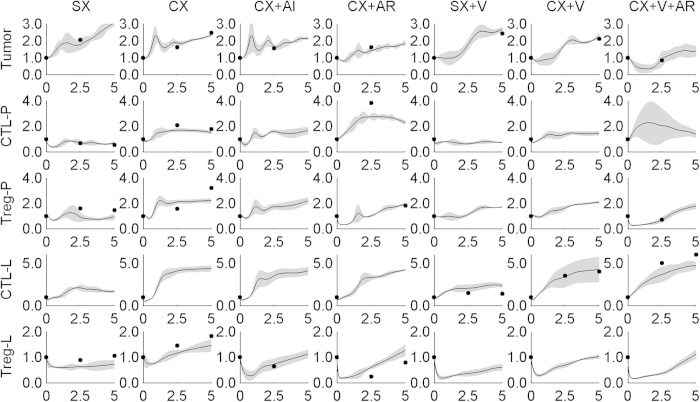
Evaluation of model performance. The plotted lines show the average dynamics from 100 replicates of simulation, and shadow areas indicate the corresponding 95% confidence intervals calculated from normal distribution statistics. For each cross-validation, one treatment was taken out as a testing sample after the model training process was completed. The black dots are the data points obtained from animal experiments. SX, sham-treated control; CX, castration; AR, Treg depletion; AI, IL-2 neutralization. In each subplot, the y-axis represents relative number of cell population and x-axis represents the time in weeks post-treatment.

Cross-validation technique is usually used for evaluating performance and accuracy of a model. We employed this technique in our study. First, we divided all of observations into two complementary subsets. One subset was used for model training and another set for model testing and validation. The goal of this study is to predict other treatment outcomes and efficacies using a mathematical modeling established through the experimental data from seven treatments conditions. An optimal way to evaluate the predictive power of a model is through cross-validation. Therefore, we decided to determine whether or not the data obtained from any of six treatments out of seven were able to accurately predict the outcomes from the remaining one. We did seven cross-validations in total (see Methods for details). In each of cross-validation, one treatment was taken out as a testing sample after the model training process was completed. In order to evaluate the stability and reliability of our model’s prediction power, the training process was repeated for 100 times with random seed in the parameter estimation algorithm. Cross-validation results are shown in Fig. 3. There was a good consistency between the prediction and experimental data and majority of the experimental data fall within 95% confidence area, indicating the predictive capability of our model was high. The overall area of the confidence region also demonstrated the stability and reliability of the model-based prediction. The confidence areas were acceptable for majority of variables except CTL-P (CTL in prostate) in the treatment group containing CX and V and AR. Note that the predictions show oscillations over time which is a common phenomenon in ODE-based modeling and the trends remain consistent with the results shown in Fig. 2B. To further confirm the predictive capability of this model, we also conducted an alternative cross-validation, in which only one set of experimental data instead of all data from one treatment condition was used as a testing sample for model prediction (Fig. S4). The predictive power was similar to that observed above. These results indicated that our model achieved high accuracy and reliability in predicting outcomes of combined treatments. Therefore, we believe that the established model has potential to be used as a tool in predicting outcomes of other treatment combinations.

### Evaluation of the model performance using tumor sizes as treatment outcomes

**Figure 4:**
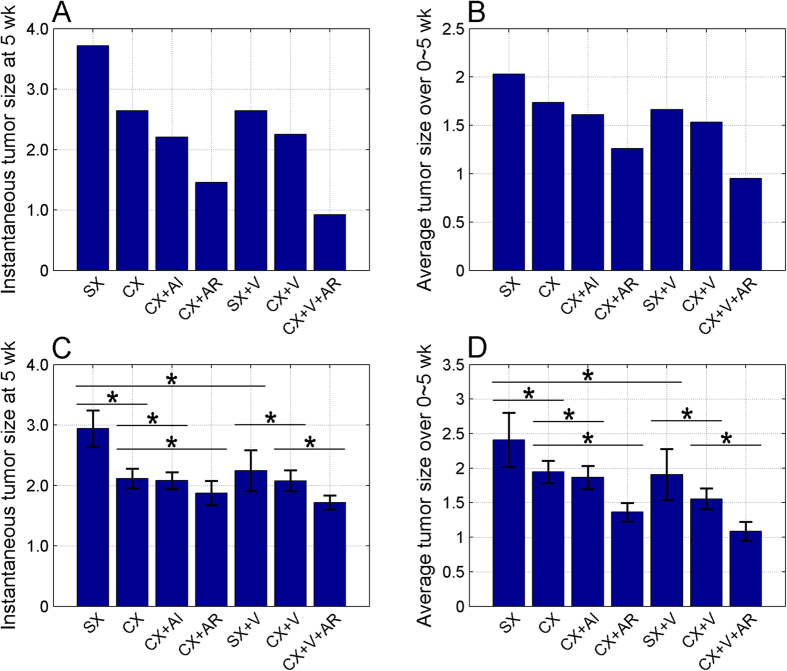
Predicting the effects of combined treatments on tumor growth. The effects of treatments on instantaneous (at 5 weeks post-treatment) and average (over 0 ~ 5 weeks post-treatment) tumor growth are showing in (**A,B**), respectively. A consistent pattern (see main text) for these two types of outcomes was demonstrated. (**C–D**) The derived pattern was confirmed with statistical significance through 100 replicates in model training process. The data are presented as mean  ± s.d. The asterisk shows the significant difference (p-value <0.05) of the comparison between the considered two conditions. The complete comparison among all seven treatment conditions are provided in Table S3. The p-value was calculated by unpaired student’s t-test with two-tailed setting. SX, sham-castration or non-treated control; CX, castration; AR, Treg depletion; AI, IL-2 neutralization.

To determine the biological significance of this model, we examined the accuracy of our model in predicting tumor size as outcomes. Two types of tumor sizes were used as treatment outcomes. One was instantaneous tumor size at 5 weeks and another one average tumor size over 0~5 weeks after treatment. Based on the established model using experimental data obtained from seven treatment conditions (Fig. 2), we calculated and compared the tumor sizes at and during 5 weeks after treatments. The changes in tumor sizes at and during 5 weeks following 7 different treatment conditions were similar as shown in Fig. 4A,B. The Pearson correlation coefficient (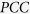) was 0.98 and p-value 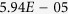, indicating a good correlation between two types of tumors. Moreover, our analysis showed two tendencies for the treatment outcomes. ADT/CX itself inhibited tumor growth. Combination of ADT with either IL-2 neutralization (AI) or Treg depletion (AR) enhanced the role of ADT. The treatment efficacy of ADT plus Treg depletion was better than that of ADT plus IL-2 neutralization. The general tendency was CX + AR > CX + AI > CX > SX (control). Immunotherapy/vaccination (V) enhanced the treatment effect of ADT and addition of Treg depletion achieved even better outcomes. The treatment efficacies with vaccination were CX + V + AR > CX + V > SX + V > SX. In order to determine if these predictions were statistically significant, we repeated model training process over 100 times. The results were shown in Fig. 4C,D. Student’s t-test indicated that the ranked efficacies for various treatments were statistically significant (p-value <0.05) in terms of two types of tumor growths (Table S3).

**Figure 5:**
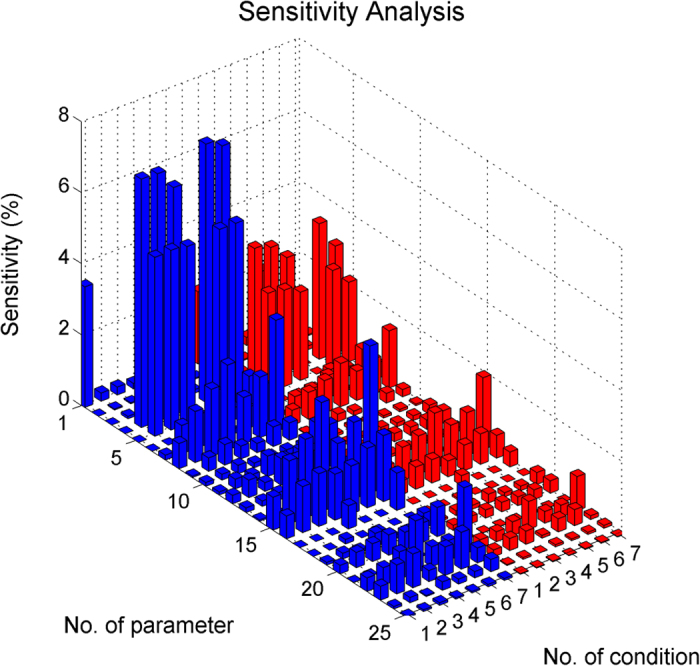
Sensitivity analysis of the model parameters. The local sensitivity (z-axis) for an individual parameter (x-axis) under a certain treatment condition (y-axis) was calculated from the established model with a 5% increase on the parameter value. The parameter is listed in Table S2. The treatment conditions are labeled as 1 (SX), 2 (CX), 3 (CX + AI), 4 (CX + AR), 5 (SX + V), 6 (CX + V) and 7 (CX + V + AR). SX, sham-treated or non-treated control; CX, castration; AR, Treg depletion; AI, IL-2 neutralization. The blue columns represent the sensitivity analysis using instantaneous tumor size as an outcome and red ones for average tumor size.

To further confirm the reliability of the established model or stability of the model-based outcome prediction, we conducted local sensitivity analysis for model parameters by measuring the impact of small perturbation (5% increasing) of individual parameters on outcomes (see Methods for detail). The dynamics of tumor sizes following parameter perturbations are shown in Fig. S5. The sensitivity of individual parameters calculated from tumor dynamics are presented in Fig. 5, showing that all parameters are within 5% sensitivity (i.e. the perturbation of outcome is not beyond 5%) except for the 4^th^ parameter 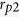 (proliferation rate of CRPC) and 5^th^ parameter 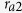 (apoptosis rate of CRPC). The sensitivity analysis confirmed the stability of established model and the model-based outcome prediction. Parameter sensitivity analysis (Fig. 5) also revealed a consistent pattern between two types of treatment outcomes (marked with blue or red colors in Fig. 5, with 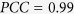 and p-value =8.0*E*–159 for the overall data).

### Model-based prediction of overall treatment efficacies

**Figure 6:**
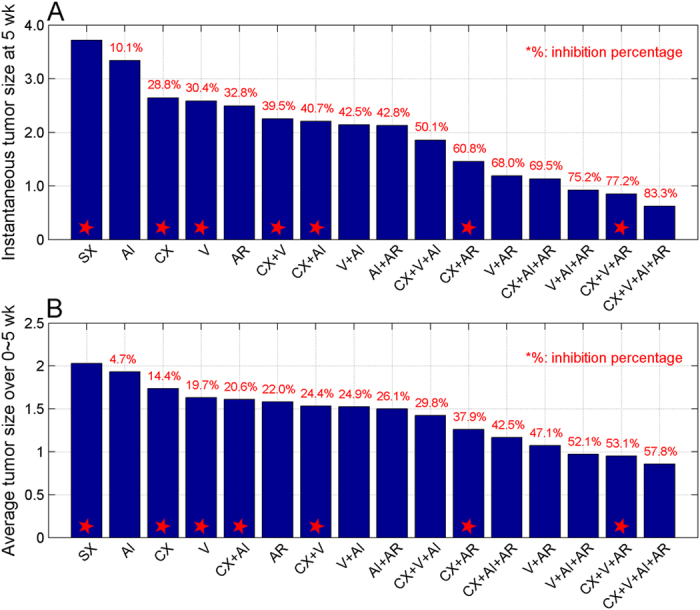
Model-based prediction of overall outcomes. The plots show the predicted treatment outcomes for all 16 possible treatment conditions, including instantaneous tumor size (**A**) and averages tumor size (**B**). The treatment conditions are listed based on their efficacies from low to high, expressed as percentage of inhibition relative to non-treated control. The predictions for the seven treatment strategies previously shown in Fig. 4 are marked with red stars. SX, sham-treated or non-treated control; CX, castration; AR, Treg depletion; AI, IL-2 neutralization.

Based on the successful evaluation of two types of treatment outcomes discussed above, now we decided to apply our established model to predict the overall outcomes for other combined treatments. We have four individual treatment conditions, including ADT, vaccination, IL-2 neutralization and Treg depletion. Combination of these 4 treatment conditions with the non-treated control group (SX) produced a total of 16 possible regimens. Seven out of 16 regimens were used in our early sections for model construction and evaluation of treatment outcomes. The remaining 9 of them had not been used so far. Taking advantage of the predictive capability of our established model, we decided to use this model to predict the treatment outcomes for all of the combined therapeutic conditions (see Methods for detail). The tumor dynamics for all 16 possible treatments are shown in Fig. S6. We calculated the predicted tumor size from 16 treatment conditions as shown in Fig. 6, Comparing the instantaneous (Fig. 6A) and average tumor size (Fig. 6B), the tumor growth inhibition pattern under 16 treatment conditions was similar. The Pearson correlation coefficient for two types of predicted outcomes were 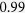 and p-value3.12*E*–13, which were consistent with our findings in the early sections. The results revealed a general order of the treatment efficacies for 16 conditions using two types of tumor size as outcomes. There were also a few inconsistencies. ADT plus IL-2 neutralization (CX + AI) ranked two positions higher and vaccination plus Treg depletion (V + AR) one position lower in the type I outcome (instantaneous tumor size) than that in the type II outcome (average tumor size). Interestingly, the predicted rank of Treg depletion was much higher than IL-2 neutralization. Among 4 treatments with a single condition, Treg depletion was ranked number 1 followed by vaccination, ADT and IL-2 neutralization. Among a total of 6 regimens with two combined therapies (CX + V, CX + AI, CX + AR, V + AI, V + AR and AI + AR), three of them contained Treg depletion (V + AR, CX + AR and AI + AR) and ranked top 3 positions as shown in Fig. 6. The remaining three were combined with IL-2 neutralization (AI + AR, V + AI and CX + AI) and ranked at the positions of 3, 4 and 5, respectively. We listed the ranking positions for of the treatments containing Treg depletion or IL-2 neutralization based on tumor-size-related outcomes in Table 1. One-tailed student’s t test (unpaired) analysis showed that Treg depletion was ranked significantly higher than IL-2 neutralization. Both Treg depletion and IL-2 neutralization have an inhibitory role of CTL, thereby to benefit therapeutic outcomes of prostate cancer treatment. The relative therapeutic efficacies of Treg depletion to IL-2 neutralization had not been investigated so far. Our analysis indicated that Treg depletion had a higher efficacy than IL-2 neutralization as an adjuvant therapy in the treatment of prostate cancer.

### Synergistic effect of Treg depletion, ADT and vaccination

**Figure 7:**
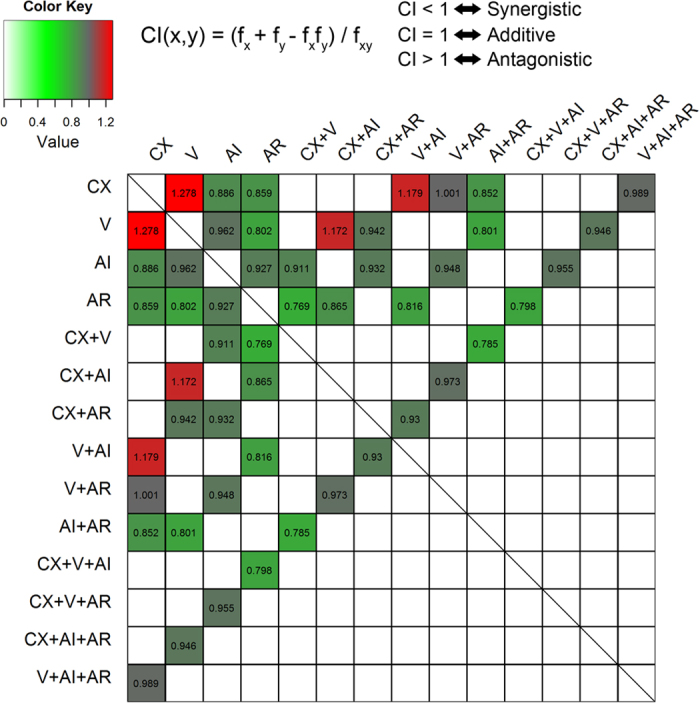
Analysis of interactions among treatments. The heatmap plot shows the symmetric matrix of Bliss combination indexes (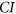
’s) for all possible treatment combinations using instantaneous tumor size as outcome. The blank boxes indicate inapplicable combinations. The value of Bliss 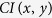 for treatment 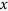 and treatment 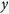 (see definition on the top of the figure) was calculated based on the individual treatment effects (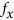 and 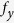) and combined treatment effect (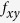). The treatment effect was defined as the percentage inhibition of instantaneous tumor size. For example, the combination of CX and V on top-left corner of the matrix has a 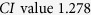 calculated by 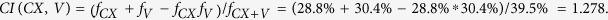

Based on the data shown above, we hypothesized that Treg depletion has a more synergistic role than IL-2 neutralization when combined with other treatments. To test our hypothesis, we assessed the synergistic effects of Treg depletion and IL-2 neutralization using tumor growth sizes as outcomes. The combined effect were evaluated using Bliss combination index (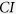) and defined *CI* < 1 as synergistic, *CI* =1 *as* additive and 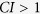 as antagonistic^33^,^34^. The index 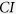 was calculated based on the model-predicted tumor inhibition rate (see Methods for detail). The 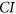 values related to the type I tumor growth for all of the combined treatment conditions were shown in Fig. 7 in a heatmap format. The 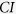 values for type II tumor growth were presented in Fig. S7. Pearson correlation test showed a very strong association between type I and Type II outcomes, with 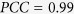 and p-value 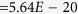. The 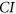 values for Treg depletion- and IL-2 neutralization-related treatments were less than 1, indicating that both Treg depletion and IL-2 neutralization had synergistic effects to other treatment conditions. We compared the 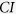 values on the 3^rd^ and 4^th^ rows of the heatmap and found that Treg depletion had a stronger synergistic impact than IL-2 neutralization (the smaller 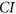 value the stronger synergy effect). Student’s t test (One-tailed and unpaired) showed that the difference was statistically significant between Treg depletion and IL-2 neutralization (p-value <0.001). More interestingly, the top three antagonistic combinations (*CI* > 1.1) were CX + V, CX + (V + AI) and (CX + AI) + V and the top three synergistic combinations (*CI* < 0.8) (CX + V) + AR, (CX + V + AI) + AR and (CX + V) + (AR + AI) (Fig. 7). Note that there is difference between the notations of A + B + C and (A + B) + C for the combined treatments. A + B + C represents three-treatment combination. The (A + B) in the (A + B) + C is counted as one treatment here, therefore, (A + B) + C represents two-treatment combination. Notice that the combination index used in this study is applicable for (A + B) + C but not for A + B + C. If we took AI out from the top three antagonistic combinations mentioned above, all of them would be converted into CX + V; similarly, the top three synergistic combinations turned into CX + V + AR. This result suggests that the combination of ADT with vaccination (CX + V) has a strong antagonistic effect and addition of Treg depletion (AR) to the combined treatment with ADT and vaccination produced a good synergistic impact. Therefore, Treg depletion is capable to reverse the antagonistic effect from combined treatment with ADT and vaccination. These findings were also supported by the synergic analysis based on the type II outcome shown in Fig. S7.

### Comparison of therapeutic efficacy and synergistic effects of four treatment approaches

**Figure 8:**
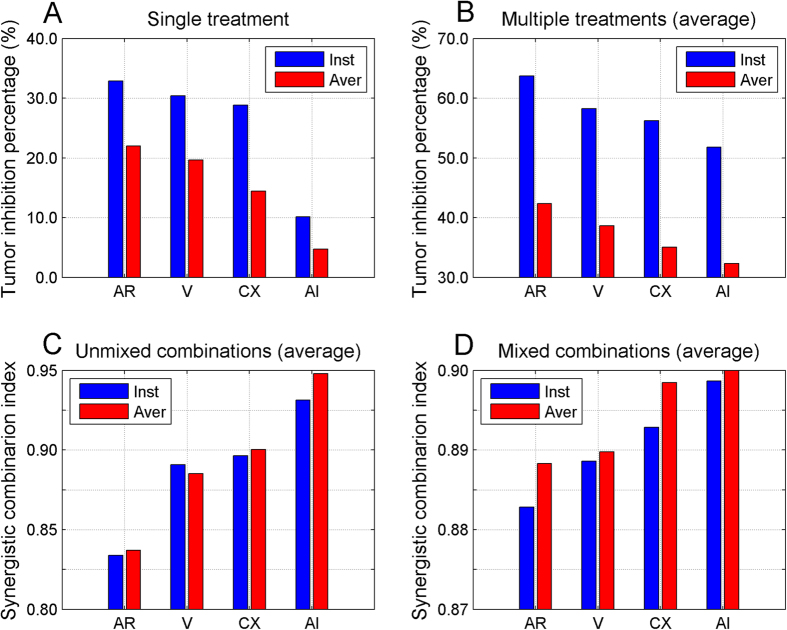
Comparison of treatment efficacies and synergistic role for individual approaches. The overall treatment efficacy for ADT (CX), vaccination (V), Treg depletion (AR) and IL-2 neutralization (AI) was plotted as percentage of tumor growth inhibition (**A–B**) and synergistic combination index (**C–D**) with a single (**A,C**) or combined treatments (**B,D**) using instantaneous or average tumor size as outcomes. For the combined treatments, the value of AR represents an average measure of total 8 AR-containing treatments, as those for ADT, CX and V. For unmixed combination index in (**C**), the value of AR is the average of 4^th^ row in Fig. 7 or Fig. S6, as those for ADT, CX and V. For mixed combination index in (**D**), the value of AR is the average of all 7 AR-related rows in Fig. 7 or Fig. S6, as those for ADT, CX and V. The blue bars represent the values calculated based on the instantaneous tumor size and the red bars on the average tumor size.

In the previous section, we have revealed that Treg depletion may have a better treatment/synergistic effect than IL-2 depletion. To further confirm if the performance of Treg depletion was better than IL-2 neutralization, as well as ADT and vaccination, we evaluated the overall effects of four types of treatments. First, we compared the effects of ADT, vaccination, Treg depletion and IL-2 neutralization, either alone or combined with other treatments, on tumor growth. As shown in Fig. 8A, the treatment efficacy in terms of percentage of tumor growth inhibition was Treg depletion (AR) > vaccination (V) > ADT (CX) > IL-2 neutralization (AI). Then we compared the average outcomes from all of the treatments containing ADT, vaccination, Treg depletion or IL-2 neutralization, respectively. For example, the Treg depletion shown in Fig. 8B represented the average of 8 Treg-containing treatments displayed in Fig. 6A. The tendency of treatment efficacies was similar to what we observed in Fig. 8A. We also compared the synergistic effects of either an individual (Fig. 8C) or average outcomes from the treatments containing ADT, vaccination, Treg depletion or IL-2 neutralization (Fig. 8D). The synergistic combination index was calculated based on *CI* values reported in Fig. 7 for type I outcome and in Fig. S7 for type II outcome (limited to the 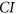
’s < 1). A smaller synergistic combination index means a better synergistic effect. As shown in Fig. 8C, the treatment with Treg depletion obtained the best synergy and followed by vaccination, ADT and then IL-2 neutralization. A same order was also seen in Fig. 8D. In summary, our model-based multiple evaluations suggested Treg depletion has a better efficacy and synergistic effect in the treatment of prostate cancer, when compared with other three treatment conditions.

## Discussion

In this study, we have used mathematical approaches to predict prostate tumor progression and treatment outcomes in a microenvironment containing immune system and other compartment. To fully characterize the impact of ADT on the immune system, a prostate-specific Pten^−/−^ mouse model was developed in our institute. A significant increase in the function of antigen-specific CD8^+^ T cells was found early after ADT and the effector function was then reduced to the same level as that in the non-castrated mice^14^. To determine the mechanism responsible for transient increase in effector function, investigation of the effect of ADT on CTL response to a well-defined immunodominant tumor antigen in non-tumor-bearing C57BL/6 mice indicated that the enhanced the proportion of antigen-specific CD8^+^ T cells in the spleen following ADT declined by 5 weeks accompanied with an increased proportion of Tregs in the ADT and immunized animals. Treg expansion was blocked in Pten^−/−^ mice by IL-2 neutralization *in vivo*. These findings indicated that ADT-induced activation of CTLs resulted in an induction of IL-2 and Treg expansion, leading to inhibition of CTLs in the prostate draining lymph nodes^15^. Therefore, our mathematical model was developed for prediction if removal of the inhibitory brakes of CTLs would improve the treatment efficacies of advanced prostate cancer in a microenvironment with multiple compartments. We predicted the outcomes from seven treatment conditions based on the *in vivo* data obtained from the prostate-specific Pten^−/−^ mouse model treated with multiple conditions. The model was trained with those experimental data. Our analysis showed that the treatment efficacy of Treg depletion was higher than that from IL-2 neutralization using tumor size as outcomes. The reason why Treg depletion is more effective than IL-2 neutralization may be due to the complicity of the interaction network topology, as well as the quantitative properties of the interactions in the proposed model shown in Fig. 1. An intuitive explanation would be that Treg depletion acts directly to reduce the Treg population; whereas IL-2 neutralization affects the Treg population indirectly. Since IL-2 neutralization will first cause a negative effect on the CD8^+^ and CD4^+^ T cell populations, as a result of that it will reduce the Tregs, Treg depletion may be preferable in reducing the regulatory effect of Tregs to complement ADT. Our mathematical model has provided a quantitative assessment for this hypothesis. Both of them have inhibitory effect on Tregs and indirectly reduce the function of CTLs. Therefore, our prediction suggests that Treg depletion is more beneficial than IL-2 neutralization does as an adjuvant or complementary to the combined immunotherapy and ADT for prostate cancer. This study provides a framework of systems biology approach in studying tumor cell-associated immune mechanisms and consequent therapeutic strategy selection.

The immune system controls cancer development by eliminating cancer cells as they develop. However, cancers can find ways to subvert the immune system. In addition, naturally occurring “brakes” in the immune system shut down anti-tumor responses. In order to design better treatment approaches for castration-resistant and metastatic diseases, it is critical to understand how anti-tumor responses develop and shut down. There are many treatment methods for cancers currently but it is virtually impossible to test the efficacy of multiple treatment methods in a time and cost-efficient manner. In order to pinpoint the best treatments with the highest likelihood of success, we have performed predictive analysis of therapeutic outcomes using the mathematical model built based on the experimental data from the Pten^−/−^ mouse model. The mathematical model took into consideration of both anti-tumor and inhibitory arms of the immune system in the tumor and lymph nodes of animals. The accuracy and robustness of the developed model was verified using cross-validation technique. Thus, this systems approach will guide the generation of better treatments for advanced prostate cancer by predicting which treatments are likely to improve cancer responses to the therapies. In the future work, we will validate our findings in the Pten^−/−^ mouse model and test our model using data obtained from human prostate cancer patients.

There are two major limitations in this current work. First, T helper cell types, especially T helper 17 cells (Th17), were not considered in the current model. We will address individual components of T help cells in response to castration in the future studies. It has been reported that Tregs and/or Th17 (rather than Th2 T cells) were involved in the development or progression of prostate cancer^35^; both CD4^+^ and CD8^+^ T cells were observed in prostate glands, and the CD4^+^ T cells consisted Th17 and Tregs cell populations^2^. Second, a limited numbers of dynamic data for lymphoid compartment were collected in the current study. Increased number of lymphoid tissue samples will be helpful in improving model accuracy in our future research.

We believe that our studies have shed lights in exploring the immune mechanisms and exploiting the effective interventions. To further understand how inhibitory pathways are generated and maintained, it is critical to identify other key molecules produced by Treg and CTL. Furthermore, pinpointing the signaling pathways activated by these interactions will reveal potential therapeutic targets. To reach this purpose, computational systems bioinformatics could be developed along with the following potential procedures: 1) generate RNA Sequencing (RNA-Seq) data and liquid chromatography–mass spectrometry (LC-MS) data from isolated immune cells, including CTLs and Tregs, in a prostate-specific Pten^−/−^ mouse model before/after castration; 2) identify the significantly enriched pathways in individual CTLs and Tregs after castration using RNA-Seq data; 3) identify the top regulated candidates in each cell type to determine cell-cell interactions and their corresponding intracellular pathways with RNA-Seq and LC-MS data; and 4) determine the signaling pathways in Tregs activated in response to IL-2 in the tumor microenvironment, and define the intracellular pathways that utilize IL-2 and the newly identified targets, by combining the enriched pathways with phosphoproteomics data analysis.

## Methods

### Ethics statement

Animal model was carried out in accordance with Wake Forest School of Medicine (WFSM), Winston Salem, NC, Animal Care and Use Committee guidelines and regulations. All experimental protocols were approved by the Institutional Animal Care and Use Committee at WFSM, Winston Salem, NC.

### Animal model and experimental data

The prostate-specific Pten^−/−^ mouse model was used in this study which has been established well in our institute^13^,^14^. This model was created many years ago^12^ and has been widely used in prostate cancer related research which mimics natural aspects of human prostate cancer development^36^. In the *in vivo* experiments^13^,^14^, total seven treatments were performed, which were respectively called in this study SX, CX, CX + AI, CX + AR, SX + V, CX + V and CX + V + AR. SX stands for sham-castration, CX for castration, V for vaccination, AI for IL-2 neutralization, AR for Treg depletion, and “+” for treatment combination. CX was treated by surgical removal of both testicles, V by injection of UV-8101-RE sarcoma cells^37^ which works similarly with FDA approved prostate cancer vaccine Provenge (or Sipuleucel-T) by programming host immune system to seek out cancer spreading in the body and attack it as if it were foreign, AI by injection of S4B6 antibody and AR by injection of PC61 antibody. All the treatments were given at the age of 14 week old for each treatment condition and then multiple variables were measured and analyzed at 16.5 week or 19 week (2.5 week or 5 week after treatment). Up to five types of variables were measured for each treatment condition, consisted of tumor weight and populations of CTL and Treg in prostate tissues and lymphoid (summary of prostate-draining lymph nodes and spleen). The tumor weight was used in our model to represent the tumor cell population (i.e., the sum of CSPC and CRPC). Using this proximate calculation is due to the following three reasons. First, the tumor weight roughly reflects the weight of total tumor cells. The immune cells infiltrating into tumor are trivial in both cell numbers and volume when compared to the tumor cells. Second, the tumor size (or tumor cell population) is proportional to the tumor weight. Third, in our mathematical model we used the relative tumor size (relative to the initial time point) but not the absolute tumor size to describe the dynamic change of the tumor over times. The animal model development and treatment protocols are given in detail in Fig. S1 and the corresponding experimental data are provided in Table S1.

### Model construction and model training

The mathematical model was constructed using ODEs to describe the dynamic interactions between tumor cells and immune cells under multiple treatment conditions. The details of equations are provided in Supplementary Text S1. In general, the model system includes 15 ODEs and 25 unknown parameters. The unknown model parameters need to be estimated through model training based on the above-mentioned experimental data. An algorithm called Modified Genetic Algorithm (MGA) was proposed to estimate those parameters by taking advantage of high computing performance (parallel computing in TACC-Texas Advanced Computing Center). The objective function minimized by MGA was defined as the sum of squared errors between simulation data and experimental data. MGA is an improved implementation of conventional GA^38^ with an additional procedure: GA parameter selection, i.e., selection of parameter settings in GA including generation number, population size, crossover probability, mutation probability, distribution index for crossover and distribution index for mutation. The reason why we have to select GA parameters is based on the fundamental fact that the GA parameters do depend on the problem to solve and there is no a universally best parameter set that you can find automatically for all problems. The procedure of parameter estimation proposed in this study is followed. First, a set of candidates (243 combinations) of GA parameters were first set up based on the empirical ranges of individual GA parameters. The candidate combinations of the GA parameters are provided in Fig. 2A. Then, for each candidate, GA was performed to obtain the fitting error. Finally, the optimal GA parameter setting was selected from the candidates through one-by-one selection of individual GA parameters by comparing each three patterns of fitting errors. After GA parameter selection was fulfilled, the final estimates of model parameters were eventually obtained by running GA one more time. The estimated parameters are provided in Table S2.

### Model parameter identifiability analysis

Coefficient of variation (CV) based on bootstrapping approach^39^ was used to study how many parameters are identifiable^16^,^40^. CV is a normalized measure of dispersion of a probability distribution of a variable, which is defined as the ratio of the standard deviation to the mean. Briefly, we first re-sampled the experimental data for 100 times using bootstrapping approach. Then, based on the re-sampled data, we obtained 100 sets of estimated parameters using the proposed optimization algorithm MGA. Finally, based on the estimated parameters sets, we calculated the CVs for all parameters and defined the number 1 as the threshold to determine the identifiability for model parameters^16^. The results for 25 model parameters are provided in Fig. S3 and Table S2.

### Model cross-validation

The cross-validation was used to evaluate how accurately our established model will perform^41^. In the cross-validation technique, the observations are first partitioned into two complementary subsets and then one subset is used to train the model and the other to test and validate the model. In this study, we performed two types of cross-validations. One is called leave one condition out cross-validation, in which we first leave out one treatment condition from total seven conditions (leave out one condition means leaving out all observations or data from that condition) the and then use the remaining conditions to predict the leave-out condition. The other is called leave one data box out cross-validation, in which we leave out only one data box (data related to one variable, up to three time points) in one condition instead of the whole data in that condition compared to the first type cross-validation. In each cross-validation, calculations were repeated 100 times (by randomly setting the search seed in GA in parameter estimation) in order to check out the stability of the model prediction. The results are provided in Fig. 3 and Fig. S4, respectively.

### Model parameter sensitivity analysis

Parameter sensitivity analysis is a tool to quantitatively determine the effect of specific parameters on the output. To understand the relationship between system responses and variations in individual model parameters, local parameter sensitivity analysis was performed in this study^42^,^43^. Briefly, we increased the estimated value for each individual parameter by 5% and then checked the response of the system outputs to determine the corresponding parameter sensitivity. The system output was defined by tumor size either instantaneous value at 5 week after treatment or the average value over 0–5 weeks after treatment. The results for 25 model parameters are provided in Fig. 5 and Fig. S5.

### Model-based synergy analysis of combined treatments

The Bliss combination index (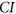) was used to evaluate the effect of combination treatment^33^,^34^. The combination index 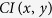 of treatment 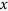 and treatment 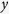 was defined based on both the individual treatment effects (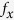 and 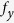) and the combined treatment effect (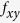), by a formula 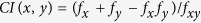. The combination treatment effect of treatment 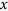 and treatment 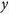 was then defined as either synergistic if 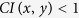 or additive if 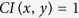 or antagonistic if 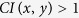. The individual treatment effect was defined by the tumor inhibition percentage based on either the instantaneous tumor size at 5 week after treatment or the average tumor size over 0–5 weeks after treatment, which are shown in Fig. 6. The results of synergy analysis for all possible combined treatments are provided in Fig. 7 and Fig. S7.

### Software

The programs for parameter estimation and all model simulations were written in the programming language C, which were performed by parallel computing in TACC-Texas Advanced Computing Center, and the results were visualized using either MATLAB software (The Mathworks, Natick, MA, USA) or RStudio software (RStudio, Boston, MA, USA). The GA implementation code was adapted from the single-objective GA code in C developed by Professor Kalyanmoy Deb’s group at Kanpur Genetic Algorithms Laboratory. The original GA source code was downloaded free charge from Prof. Deb’s lab website (http://www.iitk.ac.in/kangal/codes.shtml). The ODEs were numerically solved by using an ODE solver package DLSODE in Fortran language^44^. The DLSODE was downloaded free charge from Prof. Hindmarsh’s lab website (https://computation.llnl.gov/casc/odepack/).

## Additional Information

**How to cite this article**: Peng, H. *et al*. Prediction of treatment efficacy for prostate cancer using a mathematical model. *Sci. Rep.* **6**, 21599; doi: 10.1038/srep21599 (2016).

## Supplementary Material

Supplementary Information

## Figures and Tables

**Table 1:** AR is significantly ranked higher than AI from model-based prediction.

Treatments (candidates)	Type I outcome (Inst)		Type II outcome (Aver)	
	AR (rank)	AI (rank)	AR (rank)	AI (rank)
Single treatment (n = 4)	1	4	1	4
Two-combination (n = 6)	(1, 2, 3)	(3, 4, 5)	(1, 2, 3)	(3, 4, 6)
Three-combination (n = 4)	(1, 2, 3)	(2, 3, 4)	(1, 2, 3)	(2, 3, 4)
Statistical test	p-value = 0.0035		p-value = 0.0033	
