# Supplementary material for: Prediction of treatment efficacy for prostate cancer using a mathematical model
Source: Sci Rep. 2016 Feb 12;6:21599. doi: 10.1038/srep21599 (PMC4751505; doi:10.1038/srep21599)
Supplement: Supplementary Information [file srep21599-s1.pdf]

Supplementary data for:

Prediction of treatment efficacy for prostate cancer using a mathematical model

Huiming Peng[1], Weiling Zhao[1], Hua Tan[1], Zhiwei Ji[1], Jingsong Li[2], King Li[1], Xiaobo Zhou[1,*]

[1]Department of Radiology, Wake Forest School of Medicine, Medical Center Boulevard, Winston-Salem, NC 27157, USA

[2]College of Biomedical Engineering and Instrument Science, Zhejiang University, Hangzhou, China

[*]Corresponding author (Xiaobo Zhou): Department of Radiology, Wake Forest School of Medicine, Medical Center Boulevard, Winston-Salem, NC 27157, USA. Tel.: +1 336-713-1879; Fax: +1 336-713-5891; Email: xizhou@wakehealth.edu.

Supplementary Data Legend:

Text S1. Mathematical model

Figure S1. Animal model and experimental protocols

Figure S2. Dynamics of all variables in the model after parameter estimation, Related to Figure 2

Figure S3. Coefficient of variations of model parameters

Figure S4. Cross-validation with one data box leaving out, Related to Figure 3

Figure S5. Dynamics of tumor size following parameter perturbations, Related to Figure 5

Figure S6. Tumor dynamics in outcome prediction, Related to Figure 6

Figure S7. Synergy analysis based on type II outcome, Related to Figure 7

Table S1. Experimental data

Table S2. Model parameters

Table S3. Statistical significance of ranked efficacies, Related to Figure 4C and 4D

Text S1. Mathematical model

Here we describe the details of our model system (refer to the model diagram of Figure 1 in the main text). The mathematical model was constructed using ordinary differential equations (ODEs) to describe the dynamics of the considered variables in the system. The model considered the combined treatment of vaccination therapy and androgen deprivation therapy (surgical castration) as well as additional perturbations of IL2 neutralization and Treg depletion. The modeling techniques included the techniques that were used in modeling androgen deprivation therapy (Ideta, et al., 2008; Jain, et al., 2011; Morken, et al., 2014; Portz, et al., 2012) and the techniques that were used in modeling vaccination therapy (Agur and Vuk-Pavlovic, 2012; Kogan, et al., 2012; Kronik, et al., 2010) in prostate cancer. The model also considered the biological mechanism of interaction between CTL and Treg through secreted IL2 which have been recently reported (Akins, et al., 2010; Tang and Dubey, 2012; Tang, et al., 2012).

Vaccine (V): Equation (1) describes the dynamic of vaccine (V) in prostate compartment. To model the vaccination treatment, we introduce a variable of vaccine V, corresponding to UV-8101-RE in animal data and experiments (Akins, et al., 2010), into the model system in equation (1) while describing its function to promote mature DC in equation (7). We assume that the vaccine is injected into prostate tissue with a normalized initial value $V_0 = 1$, and then the dynamic of vaccine follows an exponential decay in equation (1), with a fixed half-life $t_{1/2} = 1$ (week) and thus a fixed decay rate $\lambda_V = ln2/t_{1/2} = 0.6931$.

$$\dot{V} = -\lambda_V V \tag{1}$$

Androgen (A): Equation (2) describes the dynamic of androgen in prostate. To model the surgical castration (CX) treatment, we follow a similar strategy used in (Ideta, et al., 2008) by introducing an indicator function 1_{CX}. On-treatment (CX) is represented by $1_{CX} = 1$, and in this case the dynamic of androgen follows an exponential decay in equation (1) with a fixed half-life 1 week and thus a fixed decay rate $\lambda_A = 0.6931$. Off-treatment (sham-castration, SX) is represented by $1_{CX} = 0$, and in this case the concentration of androgen keeps unchanged in equation (1) as $A = 1$.

$$\dot{A} = \lambda_A(1 - A) - \lambda_A 1_{CX} \tag{2}$$

IL2 antibody ($antiI$): Equation (3) describes the dynamic of IL2 antibody ($antiI$, abbr. for anti-IL2) in both prostate compartment and lymphoid compartment. We assume IL2 antibody has the same dynamic in prostate and lymphoid for simplicity. To model the IL2 neutralization treatment, we introduce a variable of IL2 antibody $antiI$, corresponding to S4B6 in animal data and experiments (Tang, et al., 2012), into the model system in equation (3) while describing its function to inhibit IL2 in equations (10) and (15). We assume that $antiI$ is injected into tissue with a normalized initial value $antiI_0 = 1$, and then the dynamics of $antiI$ follows an exponential decay in equation (3) with a fixed half-life 1 week and thus a fixed decay rate $\lambda_{antiI} = 0.6931$.

$$\dot{antiI} = -\lambda_{antiI} antiI \tag{3}$$

Treg antibody ($antiR$): Equation (4) describes the dynamic of Treg (regulatory T cell) antibody ($antiR$) in both prostate and lymphoid. We assume Treg antibody has the same dynamic in prostate and lymphoid for simplicity similar with the manner of IL2 antibody. To model the Treg depletion treatment, we introduce a variable of Treg antibody $antiR$, corresponding to PC61 in animal data and experiments (Akins, et al., 2010; Tang, et al., 2012), into the model system in equation (4) while describing its function to inhibit Treg in equations (9) and (14). We assume that $antiR$ is injected into tissue with a normalized initial value $antiR_0 = 1$, and then the dynamics of $antiR$ follows an exponential decay in equation (4) with a fixed half-life 1 week and thus a fixed decay rate $\lambda_{antiR} = 0.6931$.

$$\dot{antiR} = -\lambda_{antiR} antiR \tag{4}$$

CSPC (X_1): Equation (5) describes the dynamic of CSPC (castration-sensitive prostate cancer, X_1) in prostate. The first term on the right side of the equation represents the androgen-dependent proliferation, in which the proliferation rate is assumed to be directly proportional to androgen with a basic proliferation rate r_{p1}. The second term represents the androgen-dependent apoptosis, in which the apoptosis rate is assumed to be negatively correlated to androgen ($1 - A$, 1 is the maximal value of A) with a basic apoptosis rate r_{a1}. The third term represents the androgen-dependent mutation of CSPC to CRPC, in which the mutation rate is assumed to be negatively correlated to androgen with a basic mutation rate r_m which is followed by the strategy proposed in (Ideta, et al., 2008). The last term represents the killing by CTL, in which the killing efficacy is assumed to be directly proportional to CTL with a killing rate k_{CX}. Note that the first three terms are followed by similar strategies used in (Ideta, et al., 2008) and the last term is followed by similar strategy used in (Kogan, et al., 2012; Kronik, et al., 2010).

$$\dot{X_1} = r_{p1} A X_1 - r_{a1}(1 - A) X_1 - r_m (1 - A) X_1 - k_{CX} C_2 X_1 \tag{5}$$

CRPC (X_2): Equation (6) describes the dynamic of CRPC (castration-resistant prostate cancer, X_2) in prostate. The first term on the right side of the equation represents the proliferation, which is independent on androgen, with a constant proliferation rate r_{p2}. The second term represents the apoptosis, which is also independent on androgen, with a constant apoptosis rate r_{a2}. The third term represents the source from CSPC mutation. The last term represents the killing by CTL with the strategy same as CRPC in equation (5).

$$\dot{X_2} = r_{p2} X_2 - r_{a2} X_2 + r_m (1 - A) X_1 - k_{CX} C_2 X_2 \tag{6}$$

Mature DC (D_m): Equation (7) describes the dynamic of mature DC (dendritic cell) in prostate (D_m). The first term on the right of the equation represents the vaccine-dependent activation with a constant activation rate α_{VD}, in which the indicator function 1_V is used to indicate whether or not the vaccination treatment is involved. The second term represents the activation of DC by antigen shed from apoptotic tumor cells, a possible mechanism of tumor-caused immune resistance (Mitsiades, 2013; Sakaguchi, et al., 2009). The activation rate is not modeled with antigen but assumed to directly proportional to the apoptosis of both CSPC and CRPC with the same coefficient α_{XD}. The third term represents the migration of mature DC out of prostate tissue

with migration rate π_D. Note that the strategy of modeling mature DC used in this equation is similar with that used in (Kogan, et al., 2012; Kronik, et al., 2010).

$$\dot{D_m} = \alpha_{VD} V 1_V - \alpha_{XD}(r_{a1}(1-A)X_1 + k_{CX}C_2X_1 + r_{a2}X_2 + k_{CX}C_2X_2) - \pi_D D_m \quad (7)$$

CTL in prostate (C_2): Equation (8) describes the dynamic of CTL (cytotoxic T lymphocyte) in prostate (C_2). The first term on the right side of the equation represents the activation of CTL by mature DC in prostate tissue with an activation rate α_{DC}. The second term represents the source from CTL in lymphoid (C_1) which migrates out of lymphoid with a migration rate π_C and then join the pool of CTL in prostate with a probability p_C. The probability p_C was fixed at 0.5 in this study for reducing the burden in parameter estimation. The third term represents the inactivation of CTL by Treg in prostate, in which the inactivation efficacy is assumed to be directly proportional to Treg with an inactivation rate k_{RC}. The last term represents the natural death with a rate μ_C. Note that the strategy used in this equation is similar with that used in (Kogan, et al., 2012; Kronik, et al., 2010).

$$\dot{C_2} = \alpha_{DC} D_m + p_C \pi_C C_1 - k_{RC} R_2 C_2 - \mu_C C_2 \quad (8)$$

Treg in prostate (R_2): Equation (9) describes the dynamic of Treg in prostate (R_2). The first term on the right side of the equation represents the activation of Treg by mature DC in prostate tissue with an activation rate α_{DR}. The second term represents the source from Treg in lymphoid (R_1) which migrates out of lymphoid with a migration rate π_R and then join the pool of Treg in prostate with a probability p_R. The probability p_R was fixed at 0.5 in this study for reducing the burden in parameter estimation. The third term represents the activation of Treg with an activation rate α_{IR} by IL2 secreted from CTL in prostate, in accordance with the biological findings reported in (Akins, et al., 2010; Tang and Dubey, 2012; Tang, et al., 2012). The fourth term represents the activation of Treg by tumor cells with an activation rate α_{XR} (a same rate is assumed for both two types of tumor cells), a possible mechanism of tumor-caused immune resistance (Mitsiades, 2013; Sakaguchi, et al., 2009). The fifth term represents the natural death with a rate μ_R. The last term represents the inhibition of Treg by $antiR$ in prostate with a rate constant k_{antiRR}, in which the indicator function 1_{tregd} is used to indicate whether the Treg depletion treatment is implemented or not.

$$\dot{R_2} = \alpha_{DR} D_m + p_R \pi_R R_1 + \alpha_{IR} I_2 + \alpha_{XR}(X_1 + X_2) - \mu_R R_2 - k_{antiRR} antiR R_2 1_{tregd} \quad (9)$$

IL2 in prostate (I_2): Equation (10) describes the dynamic of IL2 in prostate (I_2). The first term on the right side of the equation represents the source of IL2 secreted from CTL in prostate with a constant rate α_{CI}, in accordance with the biological findings reported in (Akins, et al., 2010; Tang and Dubey, 2012; Tang, et al., 2012). The second term represents the natural degradation with a degradation rate μ_I. The third term represents the inhibition of IL2 by $antiI$ in prostate with a rate constant k_{antiII}, in which the indicator function 1_{il2d} is used to indicate whether the IL2 neutralization treatment is implemented or not.

$$\dot{I_2} = \alpha_{CI} C_2 - \mu_I I_2 - k_{antiII} antiI I_2 1_{il2d} \quad (10)$$

Functional DC (D_C): Equation (11) describes the dynamic of functional DC (D_C) in lymphoid. The first term on the right side of the equation represents the source of functional DC from mature DCs in prostate. Mature DCs migrate from prostate into lymphoid with a migration rate π_D, and then join the pool of functional DC with a probability p_D. The probability p_D was fixed at 0.5 in this study for reducing the burden in parameter estimation. The second term represents the transformation of functional DCs to regulatory DCs (D_R) with a constant rate $\alpha_{D_C D_R}$. As reported in literature, after interaction with effector cells, functional DCs become “exhausted” and give rise to a regulatory DC population (Kajino, et al., 2007; Langenkamp, et al., 2000). Note that the strategy used in this equation is same with that used in (Kogan, et al., 2012; Kronik, et al., 2010).

$$\dot{D_C} = p_D \pi_D D_m - \alpha_{D_C D_R} D_C \tag{11}$$

Regulatory DC (D_R): Equation (12) describes the dynamic of regulatory DC (D_R) in lymphoid. The first term on the right side of the equation represents the source from functional DCs described in equation (11). The second term represents the natural death with a rate μ_D. Note that the strategy used in this equation is same with that used in (Kogan, et al., 2012; Kronik, et al., 2010).

$$\dot{D_R} = \alpha_{D_C D_R} D_C - \mu_D D_R \tag{12}$$

CTL in lymphoid (C_1): Equation (13) describes the dynamic of CTL in lymphoid (C_1). The first term on the right side of the equation represents the source through activation by functional DC in lymphoid with an activation rate α_{DC}. The second term represents the natural death with a rate μ_C. The third term represents the inactivation of CTL by Treg in lymphoid, in which the inactivation efficacy is assumed to be directly proportional to Treg with an inactivation rate k_{RC}. The last term represents the migration of CTL out of lymphoid with a migration rate π_C. Note that three rates for CTL in lymphoid presented in this equation, α_{DC} and μ_C and k_{RC}, are assumed to be the same with the corresponding rates for CTL in prostate tissue (C_2) presented in equation (8) due to the same biological mechanisms.

$$\dot{C_1} = \alpha_{DC} D_C - \mu_C C_1 - k_{RC} R_1 C_1 - \pi_C C_1 \tag{13}$$

Treg in lymphoid (R_1): Equation (14) describes the dynamic of Treg in lymphoid (R_1). The first term on the right side of the equation represents the source of Treg through activation by regulatory DC in lymphoid with an activation rate $\alpha_{D_R R}$. The second term represents the source of Treg through activation with a rate α_{IR} by IL2 secreted from CTL in lymphoid, in accordance with the biological findings reported in (Akins, et al., 2010; Tang and Dubey, 2012; Tang, et al., 2012). The third term represents the natural death with a rate μ_R. The fourth term represents the migration of Treg out of lymphoid with a migration rate π_R. The last term represents the inhibition of Treg by $antiR$ in lymphoid with a rate constant k_{antiRR}, in which the indicator function 1_{tregd} is used to indicate whether the Treg depletion treatment is implemented or not. Note that all the rates for Treg in lymphoid presented in this equation except the activation rate $\alpha_{D_R R}$ and migration rate π_R are assumed to be the same with the corresponding rates for Treg in prostate tissue (R_2) presented in equation (9) due to the same biological mechanisms.

$$\dot{R_1} = \alpha_{D_R R} D_R + \alpha_{IR} I_1 - \mu_R R_1 - \pi_R R_1 - k_{antiRR} antiRR_1 1_{tregd} \tag{14}$$

IL2 in prostate (I_1): Equation (15) describes the dynamic of IL2 in lymphoid (I_1). The first term on the right side of the equation represents the source of IL2 secreted from CTL in lymphoid with a constant rate α_{CI}, in accordance with the biological findings reported in (Akins, et al., 2010; Tang and Dubey, 2012; Tang, et al., 2012). The second term represents the natural degradation with a degradation rate μ_I. The third term represents the inhibition of IL2 by $antiI$ in lymphoid with a rate constant k_{antiII}, in which the indicator function 1_{il2d} is used to indicate whether the IL2 neutralization treatment is implemented or not. Note that all the three rates for IL2 in lymphoid presented in this equation are assumed to be the same with the corresponding rates for IL2 in prostate tissue (I_2) presented in equation (10) due to the same biological mechanisms.

$$\dot{I_1} = \alpha_{CI} C_1 - \mu_I I_1 - k_{antiII} antiII_1 1_{il2d} \tag{15}$$

Finally the model system includes total 15 ODEs and 25 unknown parameters. The unknown model parameter should be estimated based upon the experimental data presented in Materials and Methods in the main text and Table S1. The summary of the estimated parameters are provided in Table S2.

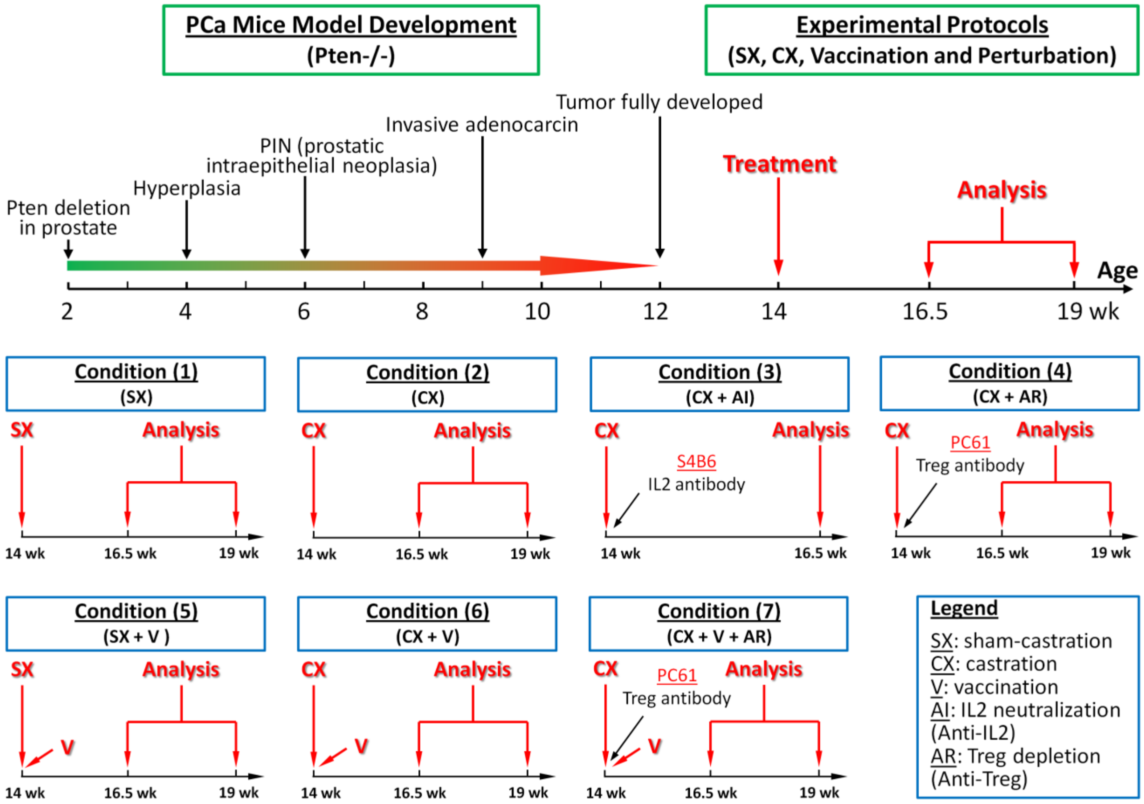

Figure S1. Animal model and experimental protocols

Upper panel shows the development of animal model and the basic experimental protocol for the timing of treatments and analysis. The lower panel shows the detailed protocols of total seven treatment conditions. Legend is shown on the bottom-right corner. The measured experimental data is shown in Table S2 and Figure 2B in the main text.

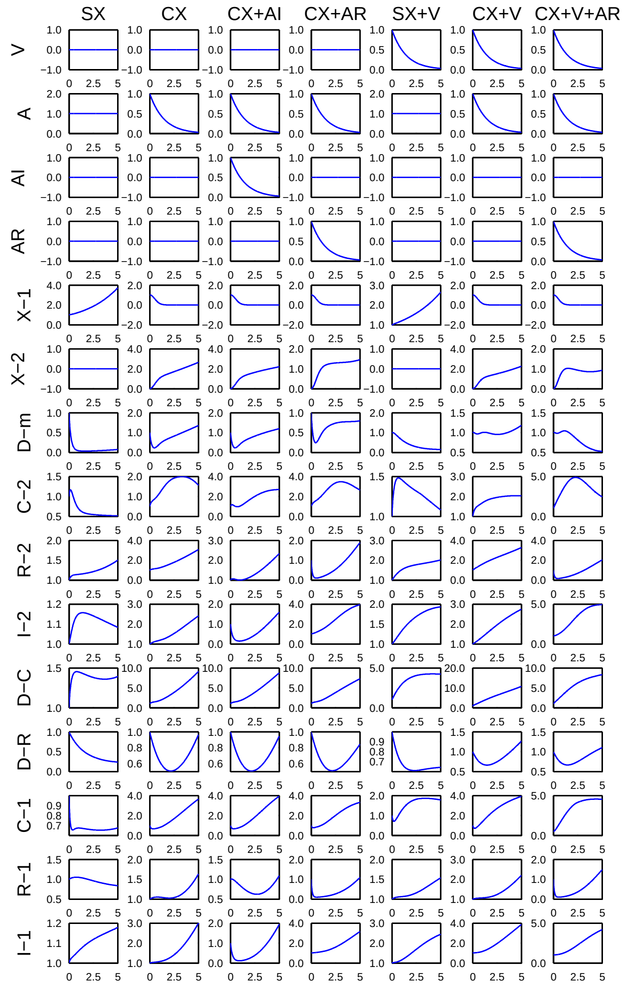

Figure S2. Dynamics of all variables in the model after parameter estimation, Related to Figure 2

Rows indicate total 15 variables in the model system (refer to Text S1). Columns indicate total 7 experimental conditions (refer to Figure S1). In each subplot, the blue curve indicate the dynamic of the corresponding variable under the indicated experimental condition. X-axis indicates the time (in weeks) post treatment and y-axis indicates the relative value of the variable in the model. The dynamics were predicted from the developed model equipped with the estimated parameters. The fitting results for the variables with experimental data are provided in Figure 2 in the main text.

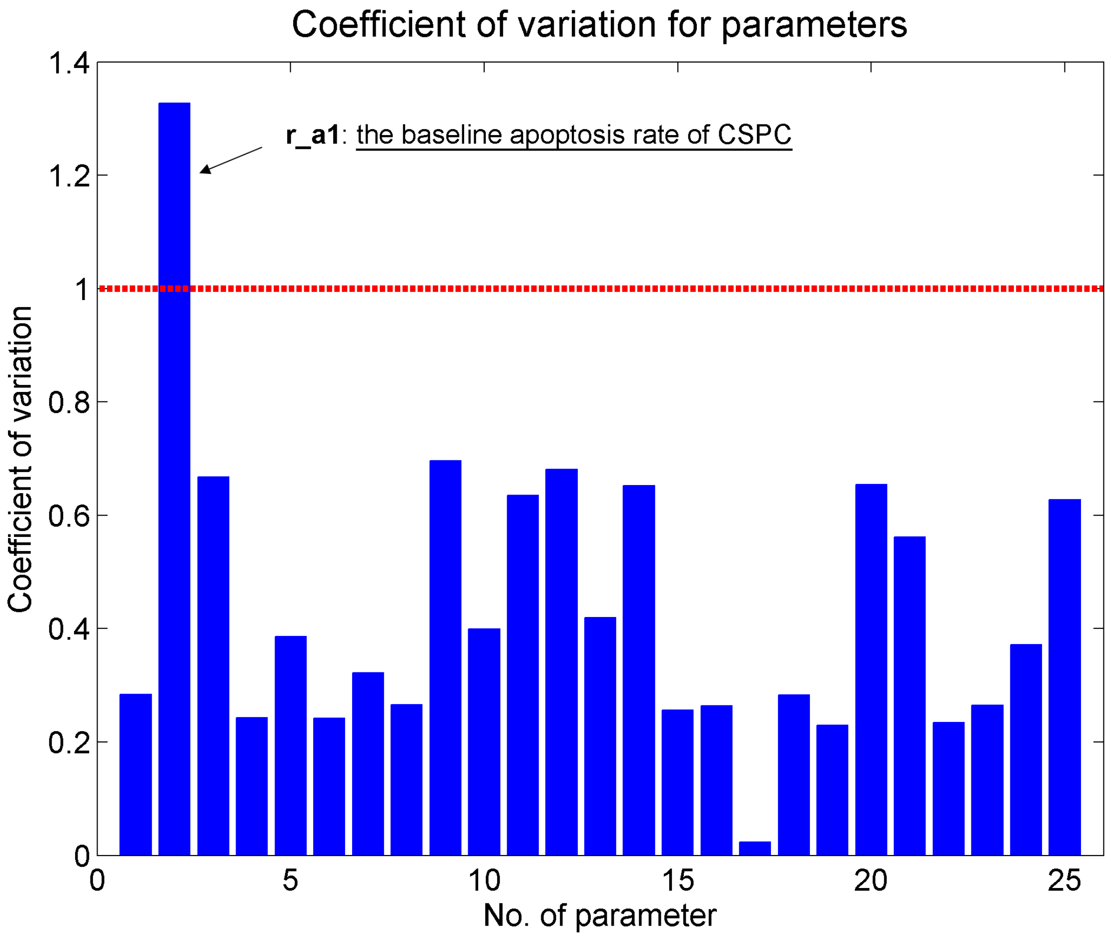

Figure S3. Coefficient of variations of model parameters

X-axis indicates the indexes of the estimated model parameters (refer to Table S1). Y-axis indicates the calculated CVs of the parameters. The red dash-line indicates the common threshold below which the parameters are identifiable. The result demonstrates that only one out of 25 parameters was non-identifiable. The original data are provided in Table S1.

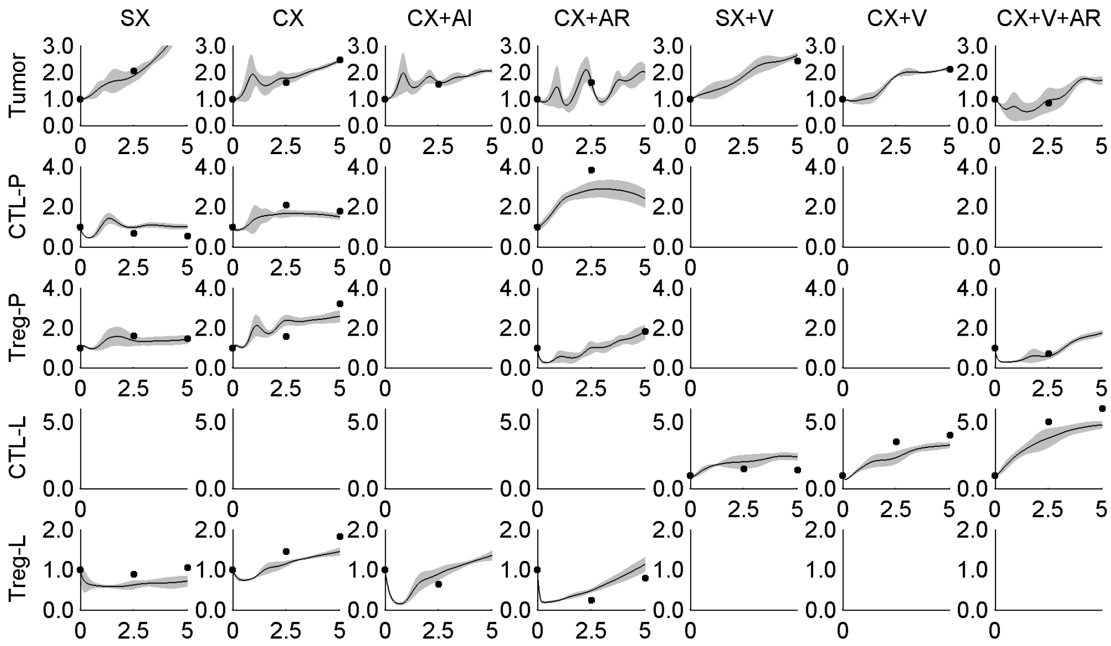

Figure S4. Cross-validation with one data box leaving out, Related to Figure 3

The plots show 21 cross-validation results regarding 21 experimental data boxes. One data box means two or three dynamic data points of one variable under a certain experimental condition. Each subplot shows one cross-validation result by leaving out the corresponding data box from model training process. The black dots are normalized experimental data. The curves are the average dynamics from 100 replicates of the simulation, and the shadows are the corresponding 95% confidence intervals calculated from normal distribution statistics. The blank subplots represent the case of no experimental data. X-axis indicates the time (in weeks) post treatment and y-axis indicates the relative value of the population size of the variable. The result for another type of cross-validation with one condition leaving out is shown in Figure 3 in the main text.

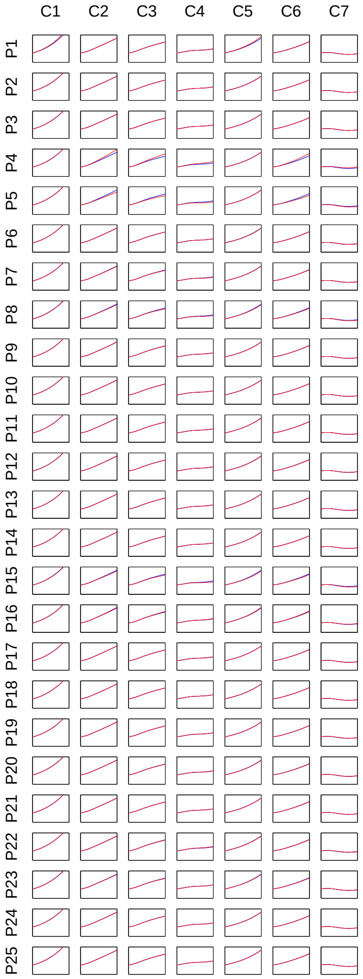

Figure S5. Dynamics of tumor size following parameter perturbations, Related to Figure 5

Rows indicate the model parameters and the order refers to Table S1. Columns indicate experimental conditions (refer to Figure S1). In each subplot, blue curve indicates the tumor dynamic without parameter perturbation (control) and red curve indicates the tumor dynamic with parameter perturbation (5% increase). X-axis indicates the time period from 0 to 5 weeks post treatment and y-axis indicates the relative value of the tumor size. Based on this result, the parameter sensitivities were calculated which are shown in Figure 5 in the main text.

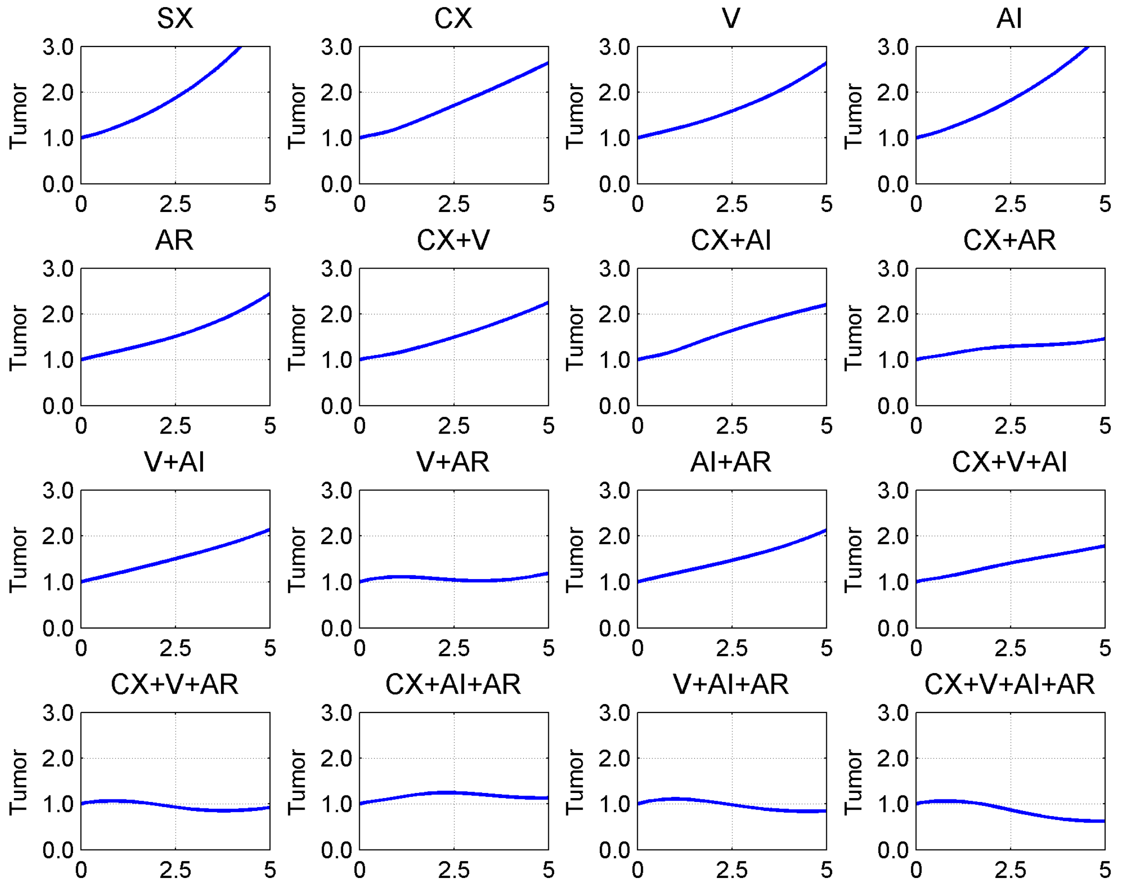

Figure S6. Tumor dynamics in outcome prediction, Related to Figure 6

The plots show the predicted dynamics of the tumor size under all 16 possible treatment condition considered in this study using the established model. Each subplot corresponds to one treatment condition. The blue curve indicates the predicted dynamic of the tumor size. X-axis indicates the time (in weeks) post treatment and y-axis indicates the relative value of the tumor size. Based on this result, two types of treatment outcomes regarding instantaneous tumor size or average tumor size were calculated which are shown in Figure 6 in the main text.

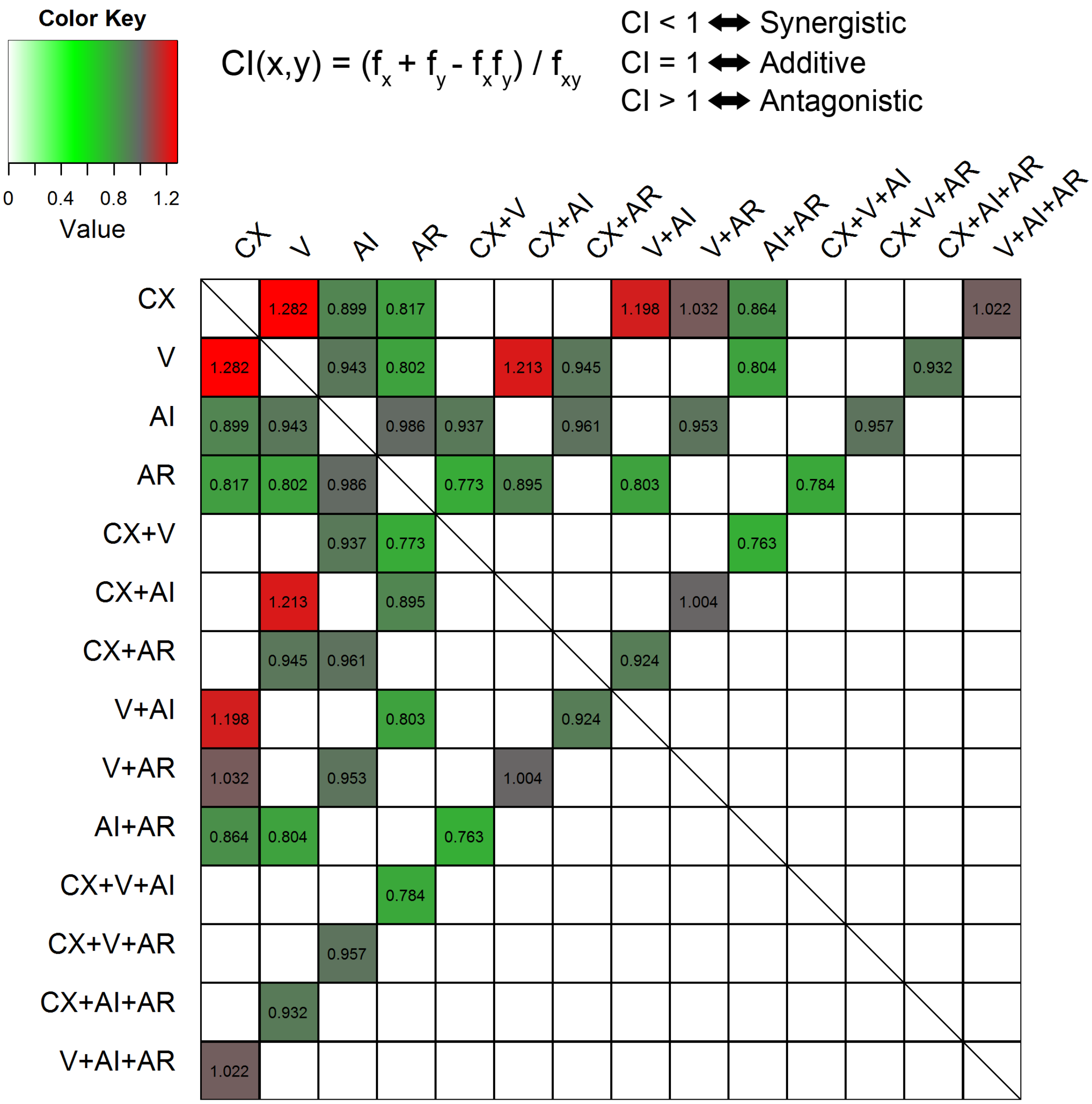

Figure S7. Synergy analysis based on type II outcome, Related to Figure 7

The heatmap plot shows the symmetric matrix of Bliss combination indexes (CI's) for all possible treatment combinations based on average tumor size as treatment outcome to evaluate the combination effects. The blank boxes indicate inapplicable combinations. The value of Bliss $CI(x, y)$ for treatment x and treatment y (see definition on the top of the figure) was calculated based on the individual treatment effects (f_x and f_y) and the combined treatment effect (f_{xy}), and the treatment effect was defined as the inhibition percentage of instantaneous tumor size which can be obtained from Figure 6B in the main text. For example, the combination of CX and V on top-left corner of the matrix was shown antagonistic effect with $CI = 1.282$ which was calculated by

$$CI(CX, V) = (f_{CX} + f_V - f_{CX} f_V)/f_{CX+V} = (14.4\% + 19.7\% - 14.4\% * 19.7\%)/24.4\% = 1.282.$$

The result of synergy analysis based on type I outcome is shown in Figure 7 in the main text.

Table S1. Experimental data

Observation / Condition		Tumor (X_1+X_2)			CTL-P (C_2)			Treg-P (R_2)			CTL-L (C_1)			Treg-L (R_1)		
		14 wk	16.5 wk	19 wk	14 wk	16.5 wk	19 wk	14 wk	16.5 wk	19 wk	14 wk	16.5 wk	19 wk	14 wk	16.5 wk	19 wk
condition 1	SX	1	2.05		1	0.68	0.54	1	1.59	1.47	1			1	0.89	1.06
condition 2	CX	1	1.61	2.46	1	2.07	1.78	1	1.56	3.21	1			1	1.45	1.82
condition 3	CX+AI	1	1.55		1			1			1			1	0.65	
condition 4	CX+AR	1	1.62		1	3.82		1		1.81	1			1	0.25	0.8
condition 5	SX+V	1		2.42	1			1			1	1.5	1.4	1		
condition 6	CX+V	1		2.11	1			1			1	3.5	4	1		
condition 7	CX+V+AR	1	0.84		1			1	0.73		1	5	6	1		

Notation for conditions

SX: sham-castration
CX: castration
V: vaccination
AI: anti-IL2 (IL2 neutralization)
AR: anti-Treg (Treg depletion)
"+" means combined treatment

Notation for observations

Tumor: CSPC and CRPC
CTL-P: CTL in prostate
Treg-P: Treg in prostate
CTL-L: CTL in lymphoid
Treg-L: Treg in lymphoid

Notation for others

1) the time-points refer to Figure S1 and the treatment was at 14 week
2) the blank means no data
3) all the data are normalized to the first time point at 14 week

Table S2. Model parameters

No	Parameter	Description	LB	UB	Estimate	CV
1	r_{p1}	Baseline proliferation rate of CSPC	0.1	10	0.323432	0.2837
2	r_{a1}	Baseline apoptosis rate of CSPC	0.1	10	0.100000	1.3273
3	r_m	Baseline mutation rate of CSPC to CRPC	0.1	10	6.702665	0.6677
4	r_{p2}	Baseline proliferation rate of CRPC	0.1	10	1.426189	0.2431
5	r_{a2}	Baseline apoptosis rate of CRPC	0.1	10	1.061691	0.3864
6	α_{VD}	Activation rate of mature DC by vaccine	0.1	10	5.000000	0.2421
7	α_{XD}	Activation rate of mature DC by tumor	0.1	10	2.096368	0.3217
8	α_{DC}	Activation rate of CTL by DC	0.1	10	3.077551	0.2657
9	α_{DR}	Activation rate of Treg by DC	0.1	10	0.739121	0.6960
10	α_{XR}	Activation rate of Treg by tumor	0.1	10	0.144144	0.3996
11	α_{CI}	Secretion rate of IL2 from CTL	0.1	10	0.408862	0.6353
12	α_{IR}	Activation rate of Treg by IL2	0.1	10	0.354526	0.6811
13	$\alpha_{D_C D_R}$	Transformation rate of functional DC to D_R	0.1	10	0.100000	0.4192
14	$\alpha_{D_R R}$	Activation rate of Treg by regulatory DC	0.1	10	0.472200	0.6526
15	k_{CX}	Killing rate of tumor by CTL	0.1	10	0.100000	0.2562
16	k_{RC}	Inactivation rate of CTL by Treg	0.1	10	1.459617	0.2636
17	k_{antiRR}	Inhibition rate of Treg by Treg antibody	0.1	10	9.999700	0.0238
18	k_{antiII}	Inhibition rate of IL2 by IL2 antibody	0.1	10	5.000000	0.2830
19	π_D	Migration rate of mature DC out of prostate	0.1	10	5.051123	0.2296
20	π_C	Migration rate of CTL out of lymphoid	0.1	10	4.111938	0.6539
21	π_R	Migration rate of Treg out of lymphoid	0.1	10	0.100000	0.5621
22	μ_C	Natural death rate of CTL	0.1	10	0.978832	0.2341
23	μ_R	Natural death rate of Treg	0.1	10	0.564691	0.2647
24	μ_I	Degradation rate of IL2	0.1	10	0.213819	0.3714
25	μ_D	Natural death rate of DC	0.1	10	0.650308	0.6278

Notation: LB, lower bound; UB, upper bound; CV, coefficient of variation
Note: the parameter whose CV value is highlighted with red color is considered to be non-identifiable

Table S3. Statistical significance of ranked efficacies, Related to Figure 4C and 4D

P-values among outcome 1
(instantaneous tumor size at 5 wk)

	CX	CX+AI	CX+AR	SX+V	CX+V	CX+V+AR
SX	6.1E-301	1.8E-322	0	1.13E-172	9.6E-370	0
CX		0.000234	3.01E-80	5.1E-14	0.000597	2.8E-239
CX+AI			7.38E-66	2.82E-22	0.944536	7.4E-233
CX+AR				2.08E-80	3.09E-58	4.77E-47
SX+V					3.35E-21	4.1E-160
CX+V						7.1E-201

P-values among outcome 2
(average tumor size over 0~5 wk)

	CX	CX+AI	CX+AR	SX+V	CX+V	CX+V+AR
SX	4.2E-104	3.1E-131	3.2E-312	2.1E-80	1.4E-244	0
CX		6.65E-14	0	0.034745	4.9E-203	0
CX+AI			4E-285	0.023373	7.7E-144	0
CX+AR				2.1E-146	1.64E-81	7.4E-159
SX+V					2.76E-72	1.7E-251
CX+V						5.9E-283

Note: 1) The left table corresponds to Figure 4C in the main text and the right table corresponds to Figure 4D in the main text; 2) The yellow color marks the pairs that have no significant difference (p-value > 0.05); 3) The p-value was calculated by unpaired student's t-test with two-tailed setting

References:

Agur, Z. and Vuk-Pavlovic, S. (2012) Personalizing immunotherapy: Balancing predictability and precision, *Oncoimmunology*, **1**, 1169-1171.

Akins, E.J., *et al.* (2010) In situ vaccination combined with androgen ablation and regulatory T-cell depletion reduces castration-resistant tumor burden in prostate-specific pten knockout mice, *Cancer Res*, **70**, 3473-3482.

Ideta, A.M., *et al.* (2008) A Mathematical Model of Intermittent Androgen Suppression for Prostate Cancer, *Journal of Nonlinear Science*, **18**, 593-614.

Jain, H.V., *et al.* (2011) Mathematical modeling of prostate cancer progression in response to androgen ablation therapy, *Proc Natl Acad Sci U S A*, **108**, 19701-19706.

Kajino, K., *et al.* (2007) Involvement of IL-10 in exhaustion of myeloid dendritic cells and rescue by CD40 stimulation, *Immunology*, **120**, 28-37.

Kogan, Y., *et al.* (2012) Reconsidering the paradigm of cancer immunotherapy by computationally aided real-time personalization, *Cancer Res*, **72**, 2218-2227.

Kronik, N., *et al.* (2010) Predicting outcomes of prostate cancer immunotherapy by personalized mathematical models, *PLoS ONE*, **5**, e15482.

Langenkamp, A., *et al.* (2000) Kinetics of dendritic cell activation: impact on priming of TH1, TH2 and nonpolarized T cells, *Nature immunology*, **1**, 311-316.

Mitsiades, N. (2013) A road map to comprehensive androgen receptor axis targeting for castration-resistant prostate cancer, *Cancer Res*, **73**, 4599-4605.

Morken, J.D., *et al.* (2014) Mechanisms of resistance to intermittent androgen deprivation in patients with prostate cancer identified by a novel computational method, *Cancer Res*, **74**, 3673-3683.

Portz, T., Kuang, Y. and Nagy, J.D. (2012) A clinical data validated mathematical model of prostate cancer growth under intermittent androgen suppression therapy, *Aip Advances*, **2**.

Sakaguchi, S., *et al.* (2009) Regulatory T cells: how do they suppress immune responses?, *Int Immunol*, **21**, 1105-1111.

Tang, S. and Dubey, P. (2012) Opposing effects of androgen ablation on immune function in prostate cancer, *Oncoimmunology*, **1**, 1220-1221.

Tang, S., *et al.* (2012) Increased CD8+ T-cell function following castration and immunization is countered by parallel expansion of regulatory T cells, *Cancer Res*, **72**, 1975-1985.
